# Genome-wide identification of ATP-binding cassette transporter B subfamily, focusing on its structure, evolution and rearrangement in ciliates

**DOI:** 10.1098/rsob.230111

**Published:** 2023-10-04

**Authors:** Xue Zhang, Yan Zhao, Weibo Zheng, Bei Nan, Jinyu Fu, Yu Qiao, Rebecca A. Zufall, Feng Gao, Ying Yan

**Affiliations:** ^1^ Institute of Evolution & Marine Biodiversity, Ocean University of China, Qingdao, Shandong 266003, People's Republic of China; ^2^ Key Laboratory of Evolution & Marine Biodiversity (OUC), Ministry of Education, Qingdao 266003, People's Republic of China; ^3^ College of Life Sciences, Capital Normal University, Beijing 100048, People's Republic of China; ^4^ School of Life Sciences, Ludong University, Yantai, Shandong 264025, People's Republic of China; ^5^ Department of Biology and Biochemistry, University of Houston, Houston, TX 77204, USA; ^6^ Laboratory for Marine Biology and Biotechnology, Laoshan Laboratory, Qingdao 266237, People's Republic of China

**Keywords:** ABCB transporter gene, gene family evolution, gene rearrangement, ciliate

## Abstract

ATP-binding cassette subfamily B (ABCB) has been implicated in various essential functions such as multidrug resistance, auxin transport and heavy metal tolerance in animals and plants. However, the functions, the genomic distribution and the evolutionary history have not been characterized systematically in lower eukaryotes. As a lineage of highly specialized unicellular eukaryotes, ciliates have extremely diverse genomic features including nuclear dimorphism. To further understand the genomic structure and evolutionary history of this gene family, we investigated the ABCB gene subfamily in 11 ciliates. The results demonstrate that there is evidence of substantial gene duplication, which has occurred by different mechanisms in different species. These gene duplicates show consistent purifying selection, suggesting functional constraint, in all but one species, where positive selection may be acting to generate novel function. We also compare the gene structures in the micronuclear and macronuclear genomes and find no gene scrambling during genome rearrangement, despite the abundance of such scrambling in two of our focal species. These results lay the foundation for future analyses of the function of these genes and the mechanisms responsible for their evolution across diverse eukaryotic lineages.

## Graphical abstract

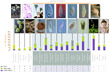

## 1. Introduction

The ATP-binding cassette transporter (ABC transporter) genes encode transmembrane proteins that can transport diverse substrates across membranes. Studies on ABC transporters have demonstrated that they are one of the largest protein superfamilies, present in both prokaryotes and eukaryotes with diverse biological functions [1]. ABC transporters in eukaryotes can be divided into full transporters or half transporters. Full transporters contain two nucleotide-binding domains (NBDs) and two transmembrane domains (TMDs) where ATP hydrolysis occurs [2,3], while half transporters consist of one NBD and one TMD, producing functional pumps by forming homo- or heterodimers [4,5].

Based on structural and functional similarity, ABC transporters are divided into eight subfamilies (A to H) [2,6]. Among them, members within the ABCB subfamily are abundant and are well known for functioning in detoxification by transporting endogenous metabolites and exogenous xenobiotics out of cells [7].

Multiple studies indicate that ABCB subfamily members may take on different functions in different systems. Despite the physiological significance of ABCB transporters in drug extrusion or pheromone export [8–12], ABCB1, ABCB4 and ABCB19 have been reported to mediate cellular transport of auxin or auxin derivatives in *Arabidopsis* [13–15], and at least two plant ABCB proteins appear to function as importers or uptake systems, a property that is very unusual for eukaryotic ABC transporters [16–18]. However, as is the case with many other gene families, studies of ABCB family members have mostly been carried out in multicellular organisms and are relatively limited in unicellular eukaryotes [19–23].

Ciliates are a highly diverse group of unicellular eukaryotes characterized by containing both a somatic macronucleus (MAC, transcriptionally active) and germline micronucleus (MIC, transcriptionally silent during vegetative growth) within one single cell [24–26]. During conjugation (the unique sexual process in ciliates), the meiotic products of the zygotic nucleus differentiate into new MAC and MIC [27]. Massive genome rearrangements occur during the development of MAC, primarily including the deletion of internally eliminated sequences (IESs) and the splicing of macronuclear destined sequences (MDSs) [26,28–31]. Of note, the extent and pattern of genome rearrangements vary dramatically among ciliates, resulting in distinct genomic features. For example, gene scrambling (the presence of fragmented sequences in non-canonical order in the MIC compared to the MAC) and alternative processing (the same MIC segments are used to generate multiple MAC sequences) are more abundant in the class Spirotrichea than Oligohymenophorea [32–38]. Therefore, the unusual nuclear dimorphism and genome-wide gene rearrangements in ciliates provide an ideal opportunity for the analysis of both the functional divergence and evolutionary characteristics of multigene families.

In the present study, we focus on the evolution and functional divergence of ABCB subfamily genes in unicellular eukaryotic ciliates. We have newly sequenced macronuclear and micronuclear ABCB subfamily genes from *Uroleptopsis citrina*, and first identified the ABCB subfamily members in the published MAC genomes of 10 representative ciliate species at the genomic level. We then studied the sequence characteristics and phylogenetic relationships of these genes within ciliates compared to other representative eukaryotes. Within ciliates, we further explored the gene duplication and genomic organization of genes in the ABCB family. These results provide novel insight into the evolution of this gene family across ciliates and set the stage for further research into their functional diversity.

## 2. Material and methods

### 2.1. Newly sequenced data

Four sequences of ABCB genes (three macronuclear nanochromosomes and the corresponding micronuclear sequences) of *Uroleptopsis citrina* were newly sequenced. The three members belonging to ABCB subfamily were identified from the draft macronuclear genome of *U. citrina* [39]. Then, we amplified and obtained the corresponding nanochromosomes using sanger sequencing. The cells of *U*. *citrina* were obtained and genomic DNA extraction was performed as described in Zheng *et al*. [39]. Primers for the three genes were designed from the shared regions, which were then used to amplify the three macronuclear sequences of ABCB genes in *U*. *citrina*. The PCR amplifications were performed using Q5 Hot Start High-Fidelity 2x Master Mix (New England BioLabs, USA) to minimize the possibility of PCR amplification errors following the optimized manufacturer's protocol. Telomere suppression PCR (TSP) was used to obtain the full-length sequence of the three ABCB subfamily genes of *U*. *citrina* [40]. The PCR primers are shown in table 1. The PCR reaction was divided into three steps. The first step used the primers DCB and gene-specific primer 1 (GSP1) with the following protocol: 7 cycles (98°C, 2 s; 72°C, 4 min), followed by 32 cycles (98°C, 2 s; 67°C, 4 min), ending with 67°C for 4 min. The second step used AP1 and GSP2 as primers with the programme, which began with 5 cycles (98°C, 25 s; 72°C, 4 min), followed by 20 cycles (98°C, 25 s; 67°C, 4 min), ending with 67°C for 4 min. Primers of the third step of PCR were AP2 and GSP3, and the procedure was the same as the second step. Thermal asymmetric interlaced PCR (TAIL PCR) was performed for amplification of micronuclear sequences [41], and the sequences of arbitrary degenerate primers used in the present work (Random-GTT, Random-GAA and Random-AGA) are shown in table 1. PCR products were purified by EasyPure Quick Gel Extraction Kit (TransGen Biotech, China) and cloned using pEASY-T1 Cloning Kit (TransGen Biotech, China), and then sequenced bidirectionally in Tsingke Biological Technology Company (Beijing, China). All contigs were assembled using SeqMan (DNASTAR).

**Table 1.** Primers for telomere suppression PCR and walking PCR.

primer	sequence (5′–3′)
DCB	GTAATACGACTCACTATAGGGCACGCGTGGTCGACGGCCCGGGCTGGTCC
	CCAAAACCCCAAAACCCCAAAA
AP1	GTAATACGACTCACTATAGGGC
AP2	ACTATAGGGCACGCGTGGT
random-GTT	NTCGASTWTSGWGTT
random-GAA	NGTCGASWGANAWGAA
random-AGA	WGTGNAGWANCANAGA

### 2.2. Available published data of ciliates

Reference genomes were downloaded from NCBI (National Center for Biotechnology Information, https://www.ncbi.nlm.nih.gov/), ParameciumDB (https://paramecium.i2bc.paris-saclay.fr/) [42] or Ciliates.org (www.ciliate.org): *Oxytricha trifallax* (GenBank assembly accession number: GCA_000295675.1, GCA_000711775.1), *Uroleptopsis citrina* (GCA_001653735.1), *Ichthyophthirius multifiliis* (GCF_000220395.1), *Paramecium biaurelia* (GCA_000733385.1), *Paramecium caudatum* (GCA_000715435.1), *Paramecium sexaurelia* (GCA_000733375.1), *Paramecium tetraurelia* strain 51 (from ParameciumDB), *Stylonychia lemnae* (GCA_000751175.1), *Tetrahymena thermophila* SB210 (GCF_000189635.1, Ciliates.org: TGD Genome Files, 1-Genome assembly.fasta), *Euplotes vannus* (Ciliates.org: EvanDB Genome Files, *Euplotes*_*vannus*_Mar2018_assembly.fasta), *Stentor coeruleus* (GCA_001970955.1).

### 2.3. Identification of ABCB subfamily genes and sequences analysis

Previously reported ABCB sequences of *Tetrahymena thermophila* were used as search queries to identify the putative ABCB genes in another 10 ciliate species. Basic Local Alignment Search Tool (BLAST) homology searches of databases were performed using Blast 2.2.31+ from the NCBI. The aligned sequences were verified one by one in NCBI blastp (https://blast.ncbi.nlm.nih.gov/Blast.cgi), with databases UniProtKB/Swiss-Prot (swissprot) and Non-redundant protein sequences (nr). The search of the open reading frame and the translation of the corresponding protein sequence of the three sequences of ABCB family in *Uroleptopsis citrina* were carried out using Augustus (http://augustus.gobics.de/).

The prediction of signal peptide and transmembrane domain was performed using the program SMART (http://smart.embl-heidelberg.de/smart/batch.pl). Collinearity analysis was performed with One Step MCScanX (Multiple Collinearity Scan) toolkit in TBtools with parameters set as follows: number of best hits = 10, evalue = 1×10^−3^, the other parameters set to default setting, to identify the collinearity of ABCBs and determine their relationships [43,44]. The resulting synteny relationships were further evaluated by CollinearScan set at an e-value of less than 1×10^−10^.

### 2.4. Determination of gene arrangement, structure and conserved motifs

The Martinez-NW method of DNASTAR MegAlign software was used to compare macronuclear and micronuclear ABCB gene sequences in order to distinguish IES and MDS. Conserved domains were identified using NCBI conserved domain search (Batch CD-Search; https://www.ncbi.nlm.nih.gov/Structure/bwrpsb/bwrpsb.cgi) and the Prosite database (https://prosite.expasy.org/). Sequences aligned to the ABCB subfamily and containing NBD structure were retained. Motif patterns of TMD and NBD in ABCB family proteins were identified using the MEME online analysis tool (http://meme.sdsc.edu/).

### 2.5. Construction of the phylogenetic tree

To elucidate the evolutionary relationship of ABCB subfamily genes, we selected 233 representative ABCB proteins from 11 ciliate species and other representative organisms (*Dictyostelium discoideum*, *Plasmodium yoelii*, *Babesia bovis*, *Saccharomyces cerevisiae*, *Arabidopsis thaliana*, *Homo sapiens* and *Mus musculus*) for phylogenetic tree construction*.* The IDs or accession identifiers of the 233 sequences are provided in electronic supplementary material, table S1. Three ABCC protein sequences of *H. sapiens, M. musculus,* and *Euplotes crassus* were chosen as the outgroup. The ABCB protein sequences were aligned using the MAFFT algorithm (MAFFT v.5) with default parameters on the GUIDANCE2 Server (http://guidance.tau.ac.il/ver2/) [45,46] and further modifications were made manually using BioEdit v.7.2.1 [47]. Maximum-likelihood (ML) analysis was performed on the CIPRES Science Gateway using RAxML-HPC2 on XSEDE v8.2.10 with the RtREV + I + G + F model (selected by ProtTest v.3.2) and 1000 bootstrap iterations [48,49]. MEGA v5.2.2 was used to visualize the tree topologies [50].

### 2.6. Detection of positive selection

Orthologous and paralogous pairs were aligned and formatted using ParaAT2.0 with the MUSCLE aligner and genetic code 6 (The Ciliate, Dasycladacean and Hexamita Nuclear Code) was selected [51]. The nonsynonymous substitution rate (Ka), synonymous substitution rate (Ks) and Ka/Ks values were calculated using KaKs_Calculator 2.0 based on the γ-YN algorithm [52]. Fisher's exact test was performed to assess the validity of the Ka and Ks values. During the calculation of Ka/Ks, the NG method was applied for *Tetrahymena thermophila*, while the GYN method was chosen for other species. A gene was considered to be under positive selection if Ka/Ks > 1 and *p*-value (Fisher) < 0.05.

## 3. Results

### 3.1. Identification and phylogenetic relationship of ABCB subfamily genes in ciliates

**Figure 1.**
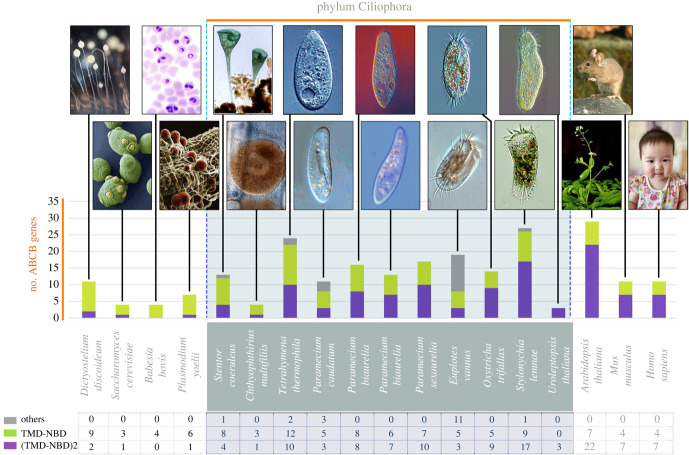
ABCB subfamily genes in 11 ciliates and other eukaryotes. ‘Others’ represent genes whose structures are not typical but contain at least one NBD domain: in *P*. *caudatum*, two genes are TMD-NBD-TMD and one gene is NBD; in *S*. *lemnae*, one gene is TMD-NBD-TMD; in *T*. *thermophila*, two genes are NBD; in *E*. *vannus*, nine genes are TMD-NBD-TMD and two genes are NBD-TMD; in *S*. *coeruleus*, one gene is TMD-NBD-TMD. *In *U*. *citrina*, only the three ABCB subfamily genes identified in this study were counted.

Through multiple cycles of BLAST and HMMER searches, we identified 137 putative ABCB transporter genes in 10 ciliates studied based on their published MAC genomes, and verified 24 ABCB genes in *Tetrahymena thermophila* (figure 1). The number of the identified ABCB transporter genes varies from four in *Ichthyophthirius multifiliis* to 27 in *Stylonychia lemnae* (figure 1).

**Figure 2.**
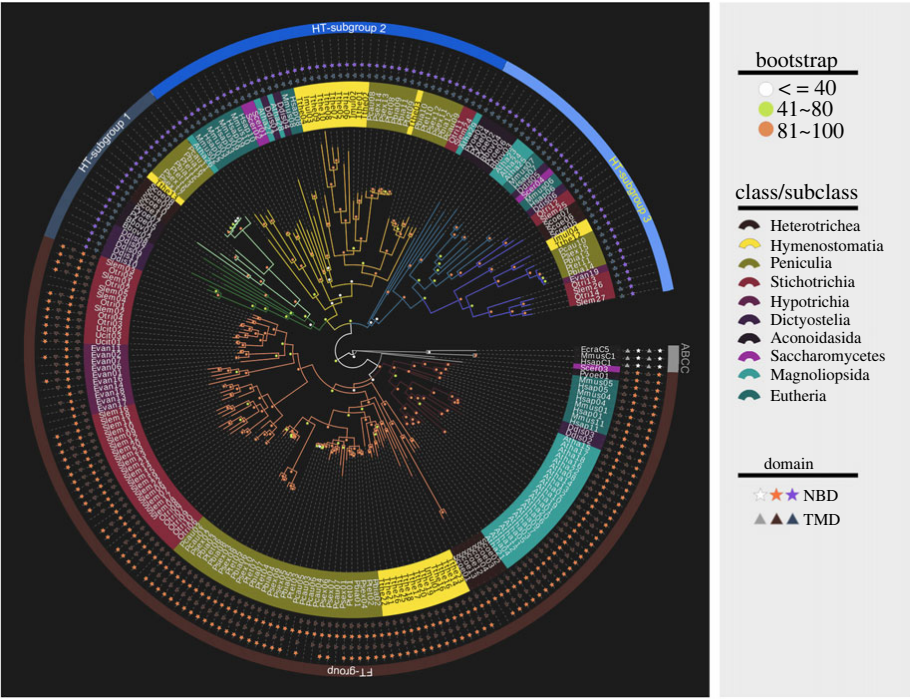
Phylogenetic relationships and domain compositions of ABCB subfamily proteins from 11 ciliate species and 7 other eukaryotes. Bootstrap values are marked with circles in different colours. The stars and triangles indicate NBD and TMD domains respectively.

In order to clarify the phylogenetic relationships of the ABCB subfamily genes in ciliates, a maximum-likelihood tree was constructed based on a complete alignment of amino acid sequences of ABCB proteins in the 11 ciliates and another seven eukaryotes (figure 2). ABCC protein sequences from *Homo sapiens, Mus musculus* and *Euplotes crassus* were used as the outgroup. In the phylogenetic trees, all the ABCB proteins were grouped into two clades, one of which containing exclusively half transporters, the other containing mostly full transporters (figure 2, blue bars with three different shades and brown bars, respectively). The group of half transporters is further divided into three subgroups (figure 2, HT-subgroup 1, HT-subgroup 2 and HT-subgroup 3). ABCB genes of *H*. *sapiens*, *M*. *musculus* and *Arabidopsis thaliana* were distributed in the group of full transporters (figure 2, FT-group) and two of the three subgroups of half transporters (figure 2, HT-subgroup 2 and HT-subgroup 3), while the other subgroup of half transporters is composed only of unicellular eukaryotes.

Generally, the phylogenetic relationships of ciliate species based on ABCB amino acid sequences are consistent with those based on ribosomal RNA genes in previous studies (figure 2) [53–55]. The group of full transporters includes 97 sequences from 11 ciliate species, and the species from the same class cluster together. For the three subgroups of half transporters, HT-subgroup 1 is composed of 21 genes, of which 12 sequences are from six ciliate species, including a single gene from *T. thermophila* (subclass: Hymenostomatia) that groups with seven genes of *Paramecium* (subclass: Peniculia), which then cluster with the four genes of *Stentor coeruleus* (class: Heterotrichea). HT-subgroup 2 is composed of 44 genes, 27 sequences of which are from eight out of the 11 ciliate species excluding *Uroleptopsis citrina*, *S*. *coeruleus* and *E*. *vannus.* With 34 sequences (including 17 sequences from 10 ciliate species) in total, HT-subgroup 3 is composed of two clades, one clade containing sequences from all 10 ciliate species, and the other consisting of sequences from *S*. *coeruleus*, *Oxytricha trifallax*, *S*. *lemnae* and other non-ciliate organisms.

### 3.2. Characteristics of gene structure and conserved motifs of the ABCB subfamily genes in ciliates

**Figure 3.**
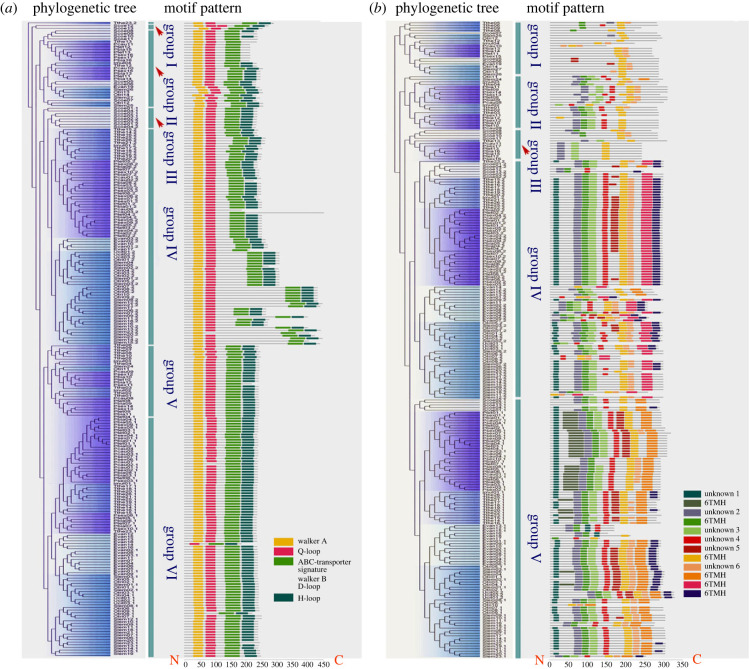
Phylogenetic relationships and architecture of conserved motifs (the NBD and TMD domain) in ABCB protein from 11 ciliates. (*a*) Mapping of the motif pattern of the NBD domain on the phylogenetic tree based on the amino acid sequences (of NBD domain). Four motifs are marked with different coloured boxes. (*b*) Mapping of the motif pattern of the TMD domain on the phylogenetic tree based on the amino acid sequences (of TMD domain). Twelve motifs are shown in different coloured boxes. Different classes/subclasses are displayed in different colours. The higher-resolution versions of phylogenetic trees corresponding to figure 3*a* and 3*b* are shown in electronic supplementary material, figure S1 and figure S2, respectively.

We next analysed the motif patterns in the ABCB genes to further elucidate the evolutionary relationships in this family. These results are consistent with the evolutionary pattern suggested by the phylogenetic analysis. The specific structural information of the protein is shown in figure 2 and electronic supplementary material, table S1. The NBD sequence region is quite conserved with almost all NBDs containing a set of characteristic motifs: Walker A, Walker B, ABC characteristic motif, H loop and Q loop (figure 3*a*).

The motif analysis of TMD shows relatively low conservation (figure 3*b*). Six of the 12 motifs analysed in TMD can be recognized as transmembrane helical (TMH) domains of ATP-binding cassette transporters. A single TMD region usually contains 2–4 TMH motifs, with a few exceptions possessing only one TMH motif (e.g. in Pbia12) or as many as five TMH motifs (e.g. in Tthe20.1) (figure 3*b*). No ciliate TMD domain was detected with all six TMH motifs in the present study. The remaining six motifs are yet of unknown classification.

The motif distribution of the two TMD domains in the same protein can also be different, indicating that this domain is likely to be related to specific substrate binding. Phylogenetic relationships of the three kinds of TMDs (TMDs.1 and TMDs.2 for full transporters and TMDs-Half for half transporters) are similar to that of NBDs: TMDs.2 (group IV) and TMDs.1 (group V) are closely related, and more distant from TMDs-Half (group I, II, III) (figure 3*b*).

### 3.3. The duplication event of ABCB subfamily in ciliates

**Figure 4.**
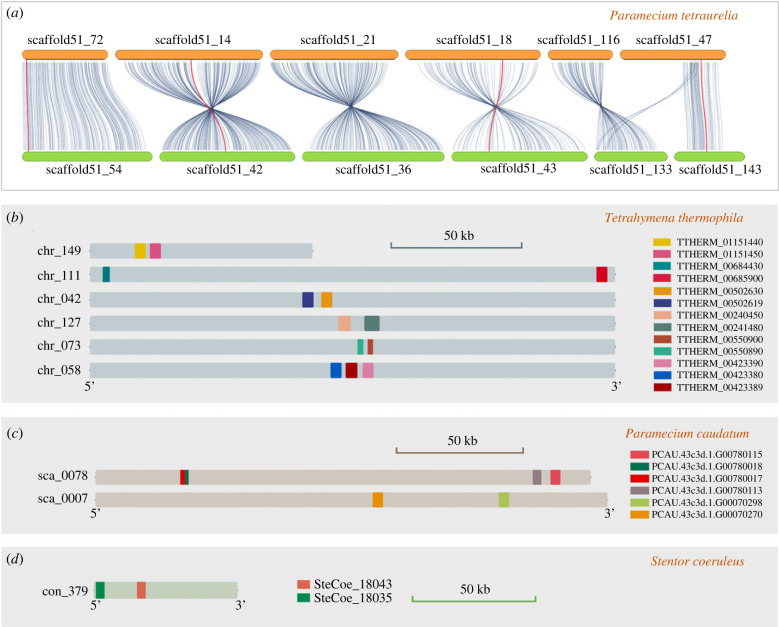
Analysis of duplication events of ABCB subfamily in ciliates. (*a*) Collinearity in *Paramecium tetraurelia*. The red and bold lines indicate the collinearity of ABCB paralogous genes with reciprocal best hits while the grey lines connect other genes that have a collinearity relationship. (*b*–*d*) ABCB genes located on the same chromosome or scaffold/contig in *Tetrahymena thermophila* (*b*), *Paramecium caudatum* (*c*) and *Stentor coeruleus* (*d*); genes are displayed in different coloured boxes. The scale bar is 50 kb.

To explore the relationship between gene duplication and the evolution of ABCB genes in ciliates, we searched for potential ABCB gene duplication events. Collinearity analysis of the ABCB genes was performed with MCScanX in *Tetrahymena thermophila*, *Ichthyophthirius multifiliis*, *Paramecium caudatum*, *Paramecium tetraurelia* (the representative of *Paramecium aurelia* complex) and *Stentor coeruleus*. *Euplotes vannus*, *Uroleptopsis citrina*, *Stylonychia lemnae* and *Oxytricha trifallax* were not included because of their extensively fragmented macronuclear genome. Thirteen ABCB genes were found in *P. tetraurelia*, each of which is located on a different scaffold. Eight of the 13 scaffolds that host ABCB genes formed four pairs through reciprocal best hit BLAST, showing strong collinearity in *P. tetraurelia* (scaffold51_72 and scaffold51_54, scaffold51_14 and scaffold51_42, scaffold51_18 and scaffold51_43, scaffold51_47 and scaffold51_143; paralogy relations are indicated by solid lines, bold for ABCB genes with a best reciprocal hit (BRH) match; figure 4*a*). Another four ABCB genes in *P. tetraurelia* can be classified as two pairs with a non-BRH syntenic match but show clear collinearity (scaffold51_21 and scaffold51_36, scaffold51_116 and scaffold51_133; figure 4*a*). No paralogous genes of the remaining one gene were detected. This result is consistent with the whole genome duplication during the evolutionary history of *P. aurelia* complex [56]. It should be noted that the lack of collinearity and gene duplication may also be caused by the differential extent of sequence contiguity of these assembled genomes.

By contrast to *P*. *tetraurelia*, analyses in *P*. *caudatum*, *T*. *thermophila* and *S*. *coeruleus* did not reveal any chromosome collinearity or gene duplication events resulting from genome duplication. However, notably, more than one ABCB gene was found on several chromosomes or scaffolds/contigs of the genomes in the three species (figure 4*b–d*). The number of ABCB genes on the same chromosomes ranges from two to four. In most cases, the ABCB genes detected on the same chromosomes are located relatively close to each other (separated by −47 bp to 13 248 bp), for example, genes in chromosomes chr_149 and chr_073 of *T*. *thermophila* (figure 4*b*). In other cases, the ABCB genes can be 45 167 to 185 245 bp apart, for example, genes in scaffold_0007 of *P*. *caudatum* and genes in chromosome chr_111 of *T*. *thermophila* (figure 4*b*–*d*).

Different from the situation in *P. tetraurelia*, tandem repeats were the main feature of ABCB gene duplication in *P*. *caudatum* and *T. thermophila*. Genes are considered to be tandem repeats if they were close in chromosomal position, with no more than five genes apart [57]. For the 24 ABCB genes found in *T*. *thermophila*, a total of eleven genes were spread in five tandem repeat gene clusters, one containing three tandem genes (figure 4*b*, chr_058) and each of the remaining four containing two tandem genes (figure 4*b*, chr_149, chr_042, chr_127, chr_073). Notably, another two of the 24 ABCB genes in *T*. *thermophila* appear to have resulted from a dispersed duplication event (figure 4*b*, genes in chromosome chr_111 of *T*. *thermophila*). For the 11 ABCB genes found in *P*. *caudatum*, four of them spread in two tandem repeat gene clusters (figure 4*c*, scaffold_0078) while two of them are in one cluster of dispersed duplication (figure 4*c*, scaffold_0007). For the 13 ABCB genes found in *S*. *coeruleus*, only two genes locate very close to each other on the chromosome, which are interrupted by four genes, and they are taken as tandem repeat genes as well (figure 4*d*, con_379). The sequence similarity of proteins in each tandem cluster ranged widely (47.8%–83.5% in *T*. *thermophila*, 60.9%–78.8% in *P*. *caudatum* and 72.8% in *S*. *coeruleus*). The sequence similarity of proteins in the one segmental duplication cluster is 22.6% in *T*. *thermophila*.

Macronuclear tandem duplicates in *T*. *thermophila*, *P*. *caudatum* and *S*. *coeruleus*, ohnologs in *P. tetraurelia*, together with micronuclear tandem duplicates in *U*. *citrina* and *O*. *trifallax* of ABCB transporters were chosen to represent relatively recent duplications. To explore the selective constraints among duplicated ABCB genes, we calculated the ratio of nonsynonymous (Ka) to synonymous (Ks) nucleotide substitutions (Ka/Ks ratio) of those 28 pairs of duplicated genes (electronic supplementary material, table S2). Our result reveals that the Ka/Ks ratio for all ABCB gene pairs is below 0.3 (mostly below 0.1), except the paralogous genes of *Scoe02* and *Scoe03* (Ka/Ks = 3.43), which resulted from gene duplication in *Stentor coeruleus*. These data indicate that ABCB gene pairs have undergone purifying selection, suggesting a highly conservative evolution of this important transporter in the gene family and their functional differentiation is limited [58]. The high Ka/Ks value of the repeated genes in *S*. *coeruleus* indicates that this pair of genes has experienced positive selection (electronic supplementary material, table S2), probably resulting in functional diversification of ABCB via adaptive evolution in *Stentor*.

### 3.4. MAC/MIC rearrangements of ABCB subfamily genes

Considering that the macronuclear genome is developed from the micronuclear genome during the sexual process, the organization of the micronuclear ABCB subfamily genes and the gene rearrangement patterns between MIC and MAC were further investigated. First, the full-length sequences of three macronuclear chromosomes containing the ABCB subfamily genes in *Uroleptopsis citrina* were obtained by traditional PCR and telomere suppression PCR (TSP PCR). The three macronuclear chromosomes are about 4600 bp in length and capped with telomeres at both ends (accession no. OP991833, OP991834, OP991835). Secondly, to assess the rearrangement between the micronucleus and macronucleus, the corresponding micronuclear sequences of the three macronuclear ABCB subfamily genes were characterized through traditional PCR and walking PCR (approx. 28.5 kb in length, accession no. OP991836).

**Figure 5.**
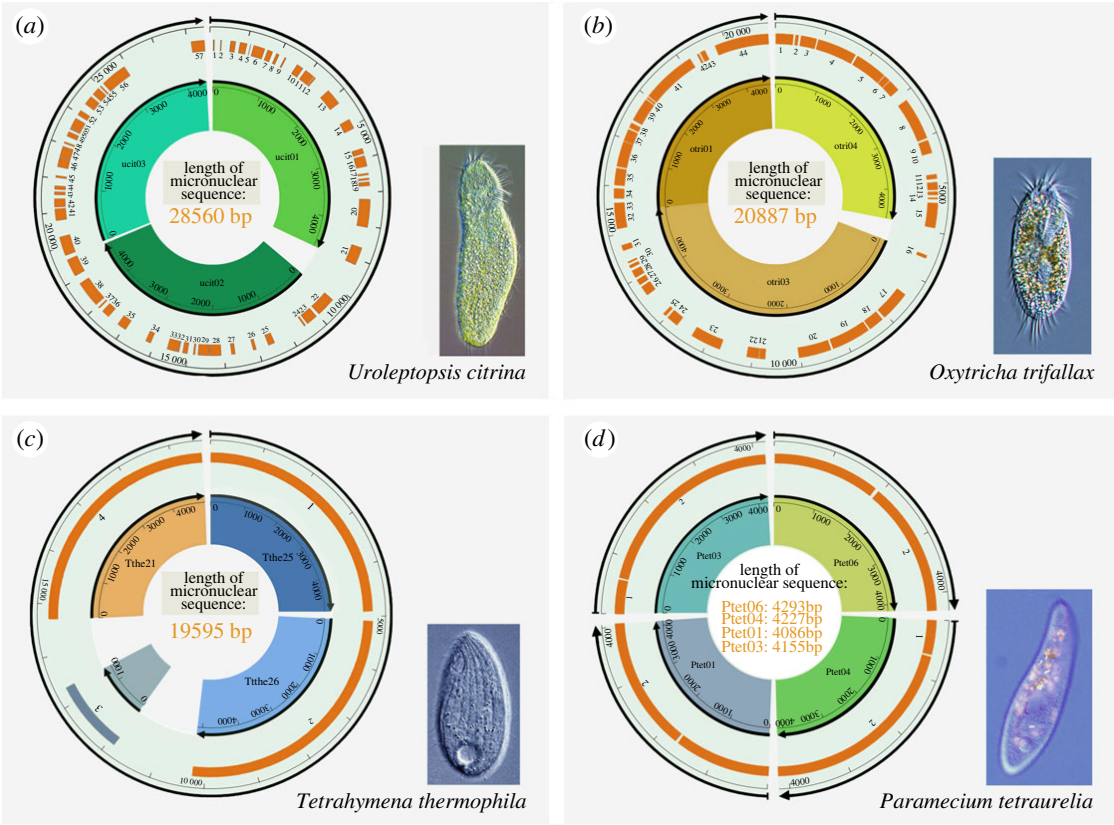
MAC/MIC rearrangements of ABCB subfamily genes in (*a*) *Uroleptopsis citrina*, (*b*) *Oxytricha trifallax*, (*c*) *Tetrahymena thermophila* and (*d*) *Paramecium tetraurelia*. The black arc with arrow in the outermost circle represents the micronuclear sequence where genes located. For each sequence, the orange bar represents MDS, and the interval between MDS is IES. The number next to the orange bar represents the serial number of the MDS. The arcs with arrows in the inner circle and the fan-shaped blocks of colour represent the macronuclear sequences where genes are located. The ruler and its numbers are used to indicate the length of each sequence. ­For *T*. *thermophila* and *P*. *tetraurelia*, the MDS region shown in the figure is the ABCB gene from the start codon to the stop codon, and it has been confirmed that there are no more MDS in this area.

Comparison of the micronuclear and macronuclear sequences reveals that the three macronuclear ABCB subfamily genes in *U*. *citrina* are assembled from a single micronuclear locus containing duplicated ABCB genes (figure 5*a*). This micronuclear locus is fragmented into 57 pieces (macronuclear destined sequences, MDSs) by internally eliminated sequences (IESs). All these MDSs are organized in order on the micronuclear locus and no scrambling was detected, relative to the macronuclear chromosome. Each ABCB gene contains 18–21 MDSs, with the length of each ranging from 5 to 819 bp. The length of the IESs also varies greatly, from 14 to 2,298 bp. The pointer sequences, i.e. pairs of identical short sequences at the adjacent MDS boundaries, are 1 to 15 bp long (electronic supplementary material, figure S3).

We also extracted the macronuclear and micronuclear ABCB gene sequences of *Oxytricha trifallax*, *Tetrahymena thermophila* and *Paramecium tetraurelia* from the published genomes, and compared the gene rearrangement patterns among the four species (figure 5). Similar to the pattern in *U*. *citrina*, the order of MDSs of the ABCB genes in the other species is the same between the macronuclear and micronuclear genomes. More specifically, in *O. trifallax*, which also has an extensively fragmented macronuclear genome, the three ABCB subfamily genes are similarly assembled from a section on the same micronuclear contig (approx. 20.9 kb in length, figure 5*b*). The micronuclear locus is fragmented into 44 MDSs, which are generally larger (31–1154 bp) than those in *U*. *citrina*, while the IESs are generally shorter (3–864 bp) compared to those in *U*. *citrina*. The length of the pointer sequences ranges from 2–12 bp.

However, the situation is slightly different in *T. thermophila* and *P. tetraurelia*. Though the three ABCB subfamily genes are also generated from a single micronuclear locus (approx. 19.6 kb in length, figure 5*c*) in *T. thermophila*, consistent with the other species, the ABCB genes are not interrupted by any IESs on the micronuclear genome, meaning that each of them is contained in a single MDS. For *P. tetraurelia*, each of the ABCB genes is fragmented into two MDSs and assembled from separate micronuclear scaffolds (figure 5*d*), which could be due to the incompleteness of the currently published micronuclear genome.

## 4. Discussion

### 4.1. Identification and phylogenetic relationship of ciliate ABCB subfamily genes

The phylogenetic relationship among the ABCB proteins revealed two groups: one containing primarily full transporters (FTs) and one with half transporters (HTs), based on ortholog clustering in a phylogenetic tree. The conservation of the ABCB domains in both major branches suggests that the genes have an ancient evolutionary origin. Furthermore, the FTs and HTs contain domains from systems with different substrate specificities [59], and are further divided into four subgroups, suggesting the possibility of functional differentiation among these groups of genes. On the whole, each group of the ABCB proteins of closely related species were clustered together and largely recapitulated the species phylogeny (figure 2). In addition, Liu *et al*. [60] reported that ABC transporters cluster depending on each gene subfamily rather than the species [60], which is consistent with our results. Therefore, the present study provides further evidence that the ABCB subfamily is ancient and suggests that the putative ancestral eukaryote already harboured complex and functionally well-differentiated ABC systems [61,62].

### 4.2. Evolution of the ABCB gene family in ciliates: gene duplication and selection patterns

The number of ABCB members in ciliates is large (figure 1), similar to some other eukaryotes, reflecting the important role of this subfamily in the life cycle of ciliates [62]. Gene duplication, resulting in gene family expansion, allows for the generation and functional differentiation of new genes. There are three main mechanisms that lead to gene duplication, namely fragment replication, tandem repeat and retrotransposition or other transposition events [4,63]. Tandem repeats occur by non-homologous recombination, which is unequal exchange during crossing over. As a result, duplicate genes formed in this way are very close in position on the chromosome. We found evidence for tandem duplication in the evolutionary pattern of six groups (13 members) of the 24 ABCB members of *Tetrahymena thermophila* (figure 4). Therefore, the replication of tandem fragments might be the primary mechanism of expansion of the *T. thermophila* ABCB subfamily. The phylogenetic relationship between *T*. *thermophila* and *Ichthyophthirius multifiliis* is very close, but the number of ABCB genes in *T*. *thermophila* is 6 times that of *I*. *multifiliis*, apparently due to gene amplification [64].

ABCB gene duplicates in *Paramecium tetraurelia* appear consistent with the history of whole genome duplication (WGD) in this species. The six groups of 12 scaffolds where 12 ABCB genes are located exhibited obvious collinearity (figure 4). The genome of *P*. *tetraurelia* has undergone at least three WGDs. An old WGD occurred before the separation of *Paramecium* and *Tetrahymena* lineages, and a more recent WGD occurred before differentiation of the *Paramecium aurelia* complex [56]. The collinearity of ABCB genes that we observed suggests that this WGD resulted in ABCB gene expansion in the *P. aurelia* complex [56,65].

Genomic analysis shows a ‘one-gene one-chromosome’ pattern in the macronuclear genomes of *Uroleptopsis citrina* and *Oxytricha trifallax* [39,66]. Therefore, we cannot gain insight into gene duplication patterns from the macronuclear sequence. However, the three ABCB genes of both of these species are tandemly distributed on the same micronuclear chromosome with similar IES-MDS structure with high sequence similarity. We thus speculate that tandem duplication was the main form of ABCB gene amplification in these species (figure 5).

In contrast to *T*. *thermophila*, two ABCB subfamily gene members were found in the same contig in *Stentor coeruleus* and *Paramecium sexaurelia*, but overall, their duplication characteristics did not provide obvious insight into the mechanism of duplication. Regardless of how the genes were duplicated, we found evidence of strong purifying selection in every comparison except one. This suggests that these genes are generally highly functionally constrained, with occasional instances of positive selection possibly leading to novel functions.

### 4.3. Genome rearrangement with ABCB gene family

Comparison between the macronuclear and micronuclear sequences of ABCB genes revealed no genetic reshuffling between genomes in the four focal ciliates. It is worth noting that the micronuclear genomes of *Oxytricha trifallax, Tetrahymena thermophila* and *Paramecium tetraurelia* exhibit distinct genome architectures. The micronuclear genome of *T. thermophila* bears IESs predominately in intergenic and intronic regions, leaving protein coding regions mostly uninterrupted [67], and only very few gene scrambling cases have been observed [68]. Our results are consistent with this pattern, as we found uninterrupted micronuclear ABCB sequences. In the micronuclear genome of *P*. *tetraurelia*, IESs can be found in both coding and noncoding regions, and there is no evidence of gene scrambling. The micronuclear sequences of ABCB subfamily genes in *P. tetraurelia* follow this pattern, with each gene contains no more than three IES intervals and no gene scrambling (four samples are shown in figure 5). In contrast, in *O. trifallax*, approximately 20% of genes do not have their fragments organized in order (i.e. they are scrambled) in the micronuclear genome [69]. As an extreme example, a dynein heavy chain family protein is separated into 245 pieces under an exceedingly complicated pattern [70]. The situation of *Uroleptopsis citrina* is similar to *O. trifallax* (figure 5) [39]. Thus, it is interesting that we find no scrambling in the ABCB genes in these species.

## 5. Summary

Our results support the hypothesis that the ABCB gene family, and ABCs more broadly, constitute an ancient gene family, likely to have already diversified in function in the earliest eukaryotes. Considering their important functions, ABCB genes have duplicated copies in the genomes of many ciliate species. While the functional relevance of these duplicates remains unclear, evidence of strong purifying selection indicates that they are under functional constraint. We expect that future studies of this and other ABC gene families will reveal more about the functions and mechanisms of selection acting on these duplicated genes.

## Data Availability

The macronuclear sequences and micronuclear sequence for ABCB genes of *Uroleptopsis citrina* have been archived on NCBI/GenBank database under the accession numbers OP991833–OP991836. The data are provided in electronic supplementary material [71].
